# Characterisation and Hypolipidaemic Effects of Tlayudas, Widely Consumed Tortillas, Containing *Ganoderma lucidum* Extracts on an In Vivo Model of Hypercholesterolaemia

**DOI:** 10.1155/ijfo/8096060

**Published:** 2025-07-29

**Authors:** Grace Preciado Iñiga, Daniel Martínez-Carrera, María E. Meneses, Miguel Sánchez, Adrián Argumedo, Myrna Bonilla, Ivan Castillo, Beatriz Petlacalco, Alfredo Morales, Nora Fernández, Wilfrido Martínez, Juan Antonio-Bautista, Mónica Sánchez-Tapia, Diana Coutiño-Hernández, Nimbe Torres, Armando R. Tovar, Hermilo Leal-Lara

**Affiliations:** ^1^Postgraduate Program on Strategies for Regional Agricultural Development (PROEDAR), Campus Puebla, College of Postgraduates (CP), Puebla, Mexico; ^2^Centre of Biotechnology of Medicinal, Functional, and Edible Mushrooms, Campus Puebla, College of Postgraduates (CP), Puebla, Mexico; ^3^SECIHTI-College of Postgraduates (CP), Puebla, Mexico; ^4^Campus Puebla, College of Postgraduates (CP), Puebla, Mexico; ^5^Department of Multidisciplinary Studies, Engineering Division, Campus Irapuato-Salamanca, University of Guanajuato, Guanajuato, Mexico; ^6^Department of Nutritional Physiology, National Institute of Medical Sciences and Nutrition Salvador Zubirán, Mexico City, Mexico; ^7^Department of Food and Biotechnology, Faculty of Chemistry, National Autonomous University of Mexico (UNAM), Mexico City, Mexico

**Keywords:** antioxidants, beta-glucans, *Ganoderma lucidum*, hypolipidaemic properties, maize, nixtamalised products, tlayudas

## Abstract

Tlayudas, a variant of tortillas, are a highly consumed and low-cost traditional Mexican food, typical of the Oaxaca region. We developed new functional food products from tlayudas (T) containing standardised hydroalcoholic extracts (T + *Gl*-1 and T + *Gl*-2) of the medicinal mushroom *Ganoderma lucidum* (*Gl*) from Mexico. These products were characterised through physicochemical and sensory properties, analysis of bioactive compounds and hypolipidaemic capacity using an in vivo model (C57BL/6 mice) of hypercholesterolaemia. Tlayudas containing *Gl* extracts are more resistant (higher cutting force) and have better sensory attributes (appearance, colour, smell, flavour, texture and acceptability). Several nutritional components and functional properties improved significantly in comparison with the control; polyunsaturated fat increased 2.3% (T + *Gl*-1 and T + *Gl*-2), total polyphenols increased from 8.2% (T + *Gl*-1) to 14.2% (T + *Gl*-2), the antioxidant capacity by oxygen radical absorbance capacity (ORAC) from 7.3% (T + *Gl*-1) to 16.5% (T + *Gl*-2) and the content of *β*-glucans from 16.6% (T + *Gl*-1) to 100% (T + *Gl*-2). The heat treatment (*ca*. 400°C for 6.40 min) of the tlayudas cooking process did not lead to denaturing of *G. lucidum* compounds, remaining bioactive and stable in the food matrix. Tlayudas containing *Gl* extracts (T + *Gl*-1 and T + *Gl*-2) decreased main serum levels of cholesterol (–21.1% and –27.5%), triglycerides (–15.1% and –25.1%) and LDL-c (–55.4% and –62.7%) in C57BL/6 mice groups studied, as well as levels of glucose (–10.4% and –31.3%) and transaminases (ALT: –40.9% and –49.7%; AST: –36.1% and –34.5%), respectively, compared with the high-cholesterol (HC) diet. Tlayudas containing the *Gl*-2 extract decreased serum lipids and glucose levels further than the atorvastatin drug. These new functional food products with hypolipidaemic properties could be used to promote healthier diets for preventing cardiovascular and chronic degenerative diseases in target populations.

## 1. Introduction

In Mexico, as centre of origin, maize is a bedrock of the cultural and biological heritage [1, 2]. Tortillas (Mexican maize cake) and other traditional food products, derived from the nixtamalised dough (maize cooked by a thermo-alkaline process and ground), are rich sources of nutrients and widely consumed. However, the nutrient content of maize products is affected by both nixtamalisation and cooking processes. Yearly intake of tortillas in Mexico has been estimated up to 79.5 kg *per capita* for rural areas, whilst 56.7 kg *per capita* for urban areas. They are particularly rich in protein, carbohydrates, fibre, calcium and vitamin E, being considered the main source of nutrients in the Mexican diet [2]. The nutritional value of tortillas can be improved by adding other foods. We increased the quality of blue maize tortillas by mixing milled basidiocarps from the edible mushroom *Pleurotus agaves* [3]. Other cases are the production of tortillas containing anthocyanins, flavonoids and saponins obtained from *Phaseolus vulgaris* [4], or the change of physicochemical properties of tortillas adding *Moringa oleifera* [5].

The nutritional and functional properties of tortillas can be improved by adding mushroom products. The medicinal mushroom *Ganoderma lucidum* contains important bioactive compounds showing immunomodulatory, anticancer, hypocholesterolaemic, hepatoprotective, antidiabetic, anti-inflammatory, antioxidant and prebiotic properties, amongst others [6–8]. The most studied compounds of this mushroom are *α*-glucans, *β*-glucans and terpenes, including ganoderic acids, as well as polyphenols and other antioxidants. These compounds show clear effects on cholesterol metabolism, through various proposed mechanisms of action. In the intestine, *β*-glucans inhibit cholesterol absorption and modulate the gut microbiota promoting bacteria that enzymatically deconjugate bile acids to increase their excretion through the expression of bile acid hydrolase activity, whilst decreasing lipid solubilisation and absorption [6]. Furthermore, oxygenosterols, ganoderols and ganoderic acids from *G. lucidum* have been shown to reduce cholesterol biosynthesis in specific metabolic routes [9–11].

Tlayudas are a traditional kind of tortilla from the central valleys of Oaxaca, Mexico [12], although they are now widely consumed elsewhere. Bioactive compounds of edible mushrooms can enrich their nutritional and functional properties. Tlayudas are large maize tortillas, 30–40 cm in diameter, browned and toasted by slow cooking on a comal (mud hotplate on a traditional oven) for improving flavour in order to get a unique food of low moisture content and long shelf life. Many rural households sell tlayudas to get additional income for the family economy. Tlayudas and edible mushrooms are part of the daily diet in rural and urban communities. However, the increasing consumption of ultraprocessed foods containing high fat and sugar content has led to a high prevalence of hypercholesterolaemia in the Mexican population. This public health problem and genetic susceptibility to dyslipidaemias [13] make cardiovascular diseases a major cause of mortality. We developed new functional food products from tlayudas containing standardised extracts from *G. lucidum*, which were characterised through their physicochemical and sensory properties, as well as their hypolipidaemic capacity using an in vivo model of hypercholesterolaemia.

## 2. Materials and Methods

### 2.1. Maize Characterisation

Kernels of local native maize (*Zea mays* L.), ‘bolita' variety cultivated in the Municipality of Santiago Matatlán, Oaxaca, according to cultural regional practices and food-grade lime purchased in a public market at Matatlán, Oaxaca, were used. Grain hardness was assessed by the flotation index (FI) indicated in the national regulation NMX-FF-034/1-SCFI-2020 [14]. The weight of 100 seeds was determined by weighing 100 grains, free of impurities. The moisture content was determined in 100 g sample using an electronic moisture metre (Delmhorst G-7, United States), according to the modified method from NMX-FF-034/1-SCFI-2020 [14]. Kernel colour was determined using a MiniScan XE Plus colorimeter (HunterLab, Virginia, United States), using the CIELAB scale to obtain the luminosity values *L*, *a*∗ and *b*∗; the hue angle (*h*∗) and chroma (*c*∗). Calculations were performed using the following formulas and methodology described by McGuire [15].

The hue was calculated as follows:

(1) $$\begin{array}{c} h=\arctan\left(\frac{a^{*}}{b^{*}}\right). \end{array}$$

The chromaticity was calculated as follows:

(2) $$\begin{array}{c} c^{*}=\sqrt{a^{{*}^{2}}+b^{{*}^{2}}}. \end{array}$$

### 2.2. Preparation of *G. lucidum* Extracts and Nutrient Composition

The Mexican strain of *G. lucidum* (Curtis) P. Karst. *sensu lato*, CP-145, is kept at the platform of genetic resources from the CB-HCFM, Mexico. It was identified at the molecular level through the ITS1-5.8S-ITS2 rDNA (GenBank, Accession Number LN998989). Cultivation, harvesting and drying of basidiocarps from *G. lucidum* (*Gl*) have previously been described [16]. For the elaboration of standardised *G. lucidum* hydroalcoholic extracts (alcohol from tequila per water 32:68, v/v), dried mushrooms were milled, macerated, extracted, concentrated (28°C–30°C, 90-110 rpm) and filter-sterilised following a patented protocol [17]. Mushroom extracts were stored at 4°C until use. *Gl*-1 are standardised extracts obtained from basidiocarps of *G. lucidum* cultivated on the control substrate (oak sawdust; *Quercus acutifolia* Née). *Gl*-2 are standardised extracts obtained from basidiocarps of *G. lucidum* cultivated on the control substrate treated with acetylsalicylic acid (ASA; 10 mM). *Gl*-1 or *Gl*-2 standardised extracts of *G. lucidum* were previously analysed according to standard protocols [6, 7, 16], showing several differences in nutrient profiles (Table S1).

Table S1 shows the chemical composition and analyses of standardised extracts *Gl*-1 and *Gl*-2 [6, 7, 16]. *Gl*-1 and *Gl*-2 extracts contained carbohydrates (0.58%), total protein (0.315%–0.365%), total dietary fibre (0.10%–0.15%), fat (0.01%), glucans (15.96%–17.01% w/w), vitamins (B1, B2, B3, B6, B12 and D), minerals (calcium, copper, iron, magnesium, manganese, phosphorus, potassium, selenium, sodium and zinc), several organic acids and an energy content of 4 kcal/100 g extract. Nutrient profiles of *Gl*-1 and *Gl*-2 extracts showed differences in total protein, total dietary fibre, total glucans, vitamins (B1, B2, B3 and B12) and minerals (copper, iron, magnesium, manganese, phosphorus, potassium, sodium and zinc).

### 2.3. Elaboration of Tlayudas and Addition of *G. lucidum* Extracts to the Maize Dough

**Figure 1:**
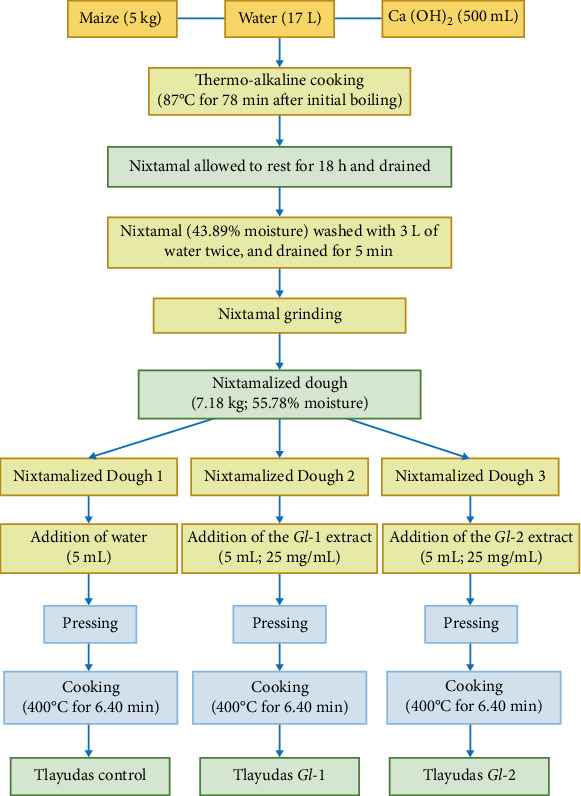
General flow diagram of the elaboration process of traditional Mexican tlayudas (control), containing standardised extracts (*Gl*-1 and *Gl*-2) from the medicinal mushroom *Ganoderma lucidum*.

The traditional process of nixtamalisation and elaboration of maize dough for tlayudas was carried out at the CB-HCFM facilities by three expert housewives from the central region of Oaxaca (Carmen Isabel Jiménez Antonio, Marilú Jiménez Santiago and Verónica Hernández Gutiérrez). A thermo-alkaline cooking of maize (*Zea mays* L.; 5 kg), water (17 L) and a Ca(OH)_2_ solution (500 mL; 1% w/v) was carried out at 87°C for 78 min and allowed to rest for 18 h, and the water derived from the nixtamalisation process (nejayote) was discarded. Thereafter, water (3 L) was used to wash the cooked maize (nixtamal; 43.89% moisture) twice and allowed to drain for 5 min. The nixtamal was ground in a mechanical mill (Molino Junior 2 HP, Arenas Tlax-Mex, Mexico), having two circular stones (35 cm diameter) and using 2 L of water in order to obtain the maize dough (7.18 kg), which was finally prepared on a stone base (metate). Immediately after preparation, 5 mL of each *G. lucidum* extract (*Gl*-1 or *Gl*-2; 25 mg/mL) was added to the traditional maize dough (100 g; 55.78% moisture) and mixed homogeneously for subsequent pressing and cooking at *ca*. 400°C for 6.40 min. Tlayudas control did not have *G. lucidum* extracts, but water instead. The general complete process for the elaboration of tlayudas is described in Figure 1. The effect of *G. lucidum* extracts on the physicochemical and sensory properties of tlayudas was determined in the following experimental final products: (1) TC (tlayudas control) = 100 g of control traditional maize dough + 5 mL of water; (2) T + *Gl*-1 (tlayudas *Gl*-1) = 100 g of traditional maize dough + 5 mL of the *Gl*-1 extract (25 mg/mL); (3) T + *Gl*-2 (tlayudas *Gl*-2) = 100 g of traditional maize dough + 5mL of the *Gl*-2 extract (25 mg/mL).

### 2.4. Physicochemical Properties

#### 2.4.1. Viscosity of Maize Doughs

Viscosity profiles of experimental doughs, control and treatments (TC, T + *Gl*-1 and T + *Gl*-2), were determined by the modified AACC method 61-02.01 [18]. Viscosity was determined using a viscometer (Brookfield model, RST-CPS, United States), at a constant speed of 400 rpm for 60 s, in the RPT-25 plate at 25°C. Data were expressed as millipascal-seconds (mPa∗s). Measurements were performed in triplicate.

#### 2.4.2. Texture Analysis of Tlayudas

The breaking force of tlayudas (hardness) was determined by the penetration force recorded during the compression of a sample using a TA-XT2 Texture Analyser (Stable Micro Systems, United Kingdom) and a cylinder (2.03 mm diameter) as the probe of the equipment at a speed of 10 mm/s and a penetration distance of 10 mm (penetration at the centre and four equidistant sides). Texture profile analysis (TPA) was carried out using the information derived from the compression of the first and second cycles using the texture analyser (values for variables fracturability, cohesiveness and chewiness obtained automatically). A sphere (6.5 mm diameter) at a speed of 0.5 mm/s was used. Measurements were performed in quintuplicate.

#### 2.4.3. Moisture in Nixtamal, Doughs and Tlayudas

Triplicate samples were placed in a drying oven 244 AD (Felisa, Jalisco, Mexico) at 100°C until constant weight was reached (*ca*. 72 h) for the determination of the moisture content, according to the standard AOAC 2000 method 920.151.

### 2.5. Bromatological Analysis of Tlayudas

Bromatological analyses were made by the specialised company Certified Laboratories Inc. (New York, United States), which is certified by the Food and Drug Administration (FDA, United States), and all its methods are standardised. Experimental tlayudas (TC, T + *Gl*-1 and T + *Gl*-2) were analysed according to standard AOAC methodologies [18]: calories (*Methods of Analysis for Nutritional Labelling*, AOAC International. 1993), calories from fat, total fat (AOAC. 2000.989.05), saturated fat (AOCS. 2002. No. Ce 1f-96), monounsaturated fat (AOCS. 2002. No. Ce 1f-96), polyunsaturated fat (AOCS. 2002. No. Ce 1f-96), trans fat (AOCS. 2002. No. Ce 1f-96), cholesterol (AOAC. 1977. No. 976.26), ash (AOAC. 2000. No. 942.05), sodium (AOAC. 2005. No. 974.14), total carbohydrate (AOAC. 2020.07), dietary fibre (AOAC. 2003. No. 985.29), total sugars (AOAC. 1995. No. 996.04), added sugars (AOAC.2020.006), protein (AOAC. 1995. No. 991.20), calcium (AOAC. 2005. No. 974.14), iron (Method CFAN/ORS/DBC/CHCB. FDA 2011), potassium (AOAC. 2005. No. 974.14) and vitamin D (AOAC. 2009. No. 982.29).

### 2.6. Bioactive Compounds in Tlayudas

Hydroalcoholic extracts (32% v/v, alcohol:water) were obtained from experimental ground tlayudas (TC, T + *Gl*-1 and T + *Gl*-2) by maceration, extraction, concentration and filter sterilisation [17].

#### 2.6.1. Determination of Total Phenolic Content

Tlayudas extracts (20 *μ*L) were mixed with 100 *μ*L of Folin and Ciocalteu's phenol reagent, adding a saturated solution of sodium carbonate (75 *μ*L) and incubating for 2 h at room temperature (22°C–25°C) in the dark. After incubation, samples were added immediately as blank, and the absorbance was measured in a 96-well microplate reader (740 nm; spectrophotometer, BioTek Epoch, United States) to determine the concentration of phenolic compounds (gallic acid equivalent, GAE), as previously described by the authors [19].

#### 2.6.2. Antioxidant Capacity

The oxygen radical absorbance capacity (ORAC) was determined following the protocol (micromole of trolox equivalent [TE] per gramme) previously described by the authors [19]. Extraction from ground tlayudas (1.0 g) was carried out using acetone per water (50:50, v/v), centrifuged at 14,000 rpm for 15 min at 4°C (Centrifuge 4-K15, Sigma, Osterode am Harz, Germany), and the supernatant was diluted with a buffer solution. The fluorescence assay including the reagent, standard preparation, stock Trolox solution and fluorescein was read in a BioTek Synergy HT plate reader (BioTek Instruments, United States).

#### 2.6.3. *β*-Glucans

Samples of ground tlayudas were used to assess the content of *β*-glucans (1→3, 1→4 linkage), according to the manufacturer's instructions of the K-BGLU kit (Megazyme, Ireland). Data were expressed as a percentage of *β*-glucans in the total weight of the sample (% w/w).

#### 2.6.4. Infrared Spectroscopy (IR) Analysis

Ganoderic acid [20] was identified in tlayudas studied by the Fourier-transformed infrared spectroscopy (FTIR) method, using a Bruker Vertex 70 model spectrometer in the attenuated total reflectance (ATR) sampling mode. The analysis was carried out taking a 5 *μ*L aliquot of each sample, reading in the spectrometer and obtaining the spectrum within the mid-infrared region (4000–400 cm^−1^). One hundred and twenty scans were taken with a resolution of 4 cm^−1^ [21]. Three measurements were made per sample, and the data were analysed using the OriginPro Version 6.1 programme (Massachusetts, United States).

### 2.7. Sensory Evaluation and Acceptability of Tlayudas

Two sensory tests were performed, using affective methods according to a 9-point structured hedonic scale, as well as an acceptance test [22]. We used the hedonic scale, 1 corresponding to ‘dislike very much', whilst 9 to ‘like very much'. The sensory evaluation tests were administered to a panel of 31 affective judges, 17 men (54.8%) ranging from 28 to 63 years old, as well as 14 women (45.2%) ranging from 24 to 60 years old. Panellists conducting the sensory evaluation were experts having at least 5 years of experience evaluating tortilla quality; they have been trained in our institution and participated in similar experimental studies assessing appearance, odour, colour, flavour and texture of tlayudas and other tortillas.

### 2.8. Effects of Tlayudas on the In Vivo Model of Hypercholesterolaemia

The effect of tlayudas studied on serum lipid levels was assessed using an in vivo model of hypercholesterolaemia. The project was evaluated and approved by the Internal Bioethics Committee (CIB) of the CB-HCFM, Campus Puebla, CP, through the registration number CIB-CB-HCFM-001. A total of 48 C57BL/6 male mice, 5 weeks old, were purchased at the National Institute of Medical Sciences and Nutrition (INCMNSZ). The animals were housed in SmartFlow ventilated racks (Tecniplast S.p.A., Italy) at standard temperature (23 ± 2°*C*), relative humidity (45%–55%) and daily cycle of light (12 h) and darkness (12 h). Mice were assigned to six different treatments (*n* = 8): (1) Ctrl: control diet (AIN-93) [23], (2) HC: high-cholesterol diet (0.5% cholesterol, Sigma-Aldrich, New Zealand), (3) HC + T: high-cholesterol diet (0.5%) + tlayudas control (60%), (4) HC + T + *Gl*-1: high-cholesterol diet (0.5%) + tlayudas containing the *Gl*-1 extract (60%), (5) HC + T + *Gl*-2: high-cholesterol diet (0.5%) + tlayudas containing the *Gl*-2 extract (60%) and (6) HC + T + At: high-cholesterol diet (0.5%) + tlayudas control (60%) + atorvastatin drug (0.03 g/100 g, Almus, Spain). Diets studied are shown in Table 1, and those including tlayudas were elaborated by mixing homogeneously all ingredients per treatment and ground tlayudas. Mice were fed ad libitum for 45 days, recording food consumption every day and body weight twice a week using an Ohaus Analytical Balance (Ohaus Corporation, New Jersey, United States). At the end of experiments, food and water were suspended for 9 h, and thereafter, mice were sacrificed by cervical dislocation. Collected blood through the portal vein was centrifuged at 1000×*g* for 10 min at 4°C to obtain the serum, which was stored at −70°C until analysis.

Cobas C111 Chemistry Analyser (Roche Diagnostics Ltd, Basel, Switzerland) was used to determine biochemical parameters in serum, according to standard analytical methods based on absorption photometry [24]. They included glucose, total triglycerides (TGs), total cholesterol, low-density lipoprotein cholesterol (LDL-c), alanine transaminase (ALT) and aspartate transaminase (AST).

### 2.9. Statistical Analysis

Three replications were performed, and data from the in vivo model were processed in the GraphPad Prism V. 8.0.2 (GraphPad Software, San Diego, California, United States) for statistical analysis, based on a completely randomised experimental design. One-way analysis of variance and Tukey's test were performed to determine statistically significant differences of experimental data (*mean* ± *standard* *error* of the mean, *p* < 0.05).

## 3. Results and Discussion

### 3.1. Characterisation of the Maize Grain

Maize grain had a FI of 38.0% (Table 2), a value similar to previous reports [25]. In Mexico, the national regulation [14] shows that the maximum hardness in maize grains for the elaboration of nixtamalised products is determined by the FI, which must be 40% as a maximum; grains have an intermediate hardness at this level. Another important parameter for nixtamalisation is the weight of 100 grains (HGW), in which samples having less than 38 g are considered small grains. In the present study, the HGW was 46.3 g (Table 2), higher than previous reports from Amador-Rodríguez et al. (38 g) [26] and Vázquez-Carrillo et al. (32.4 g) [27]. Several parameters determine the nixtamalisation quality of maize. The moisture of local grain (MG) was of 11.9%, lower than those of maize landraces (13%) commonly cultivated in Mexican highlands [27] or other Chinese maize varieties (17.8% to 24.5%) [28]; these differences were mainly due to environmental conditions and cultivation practices. The moisture content of the nixtamal should range from 36% to 42%, according to the national regulation [14]. In this study, the nixtamal had a moisture content of 43.9% (Table 2), a value lower than those recorded by Vázquez-Carrillo et al. (45.4%) [27] and Torres et al. (50.3%) [29]. Colour values recorded were *L*∗ = 68.6, *a*∗ = 4.7 and *b*∗ = 29.6. Values for *h*∗ and *c*∗ were 81.1 and 30.0, respectively. Therefore, maize grains studied showed high luminosity, close to strict yellow colour, and low saturation and orientation towards yellow (Table 2). Palacios-Pola et al. [30] reported lower *L*∗ values in three maize varieties, ranging from 62.9 to 63.8.

### 3.2. Physicochemical Properties

#### 3.2.1. Moisture and Viscosity in Doughs for the Elaboration of Tlayudas

The moisture content of maize dough from tlayudas control (TC) was significantly different from those recorded in T + *Gl*-1 and T + *Gl*-2 doughs (Table 3). The moisture ranging from 58.2% to 58.7% is lower than other research works [4, 29]. The results from viscosity of experimental doughs (Table 3) also showed significant differences. The lowest viscosity was recorded in the TC dough (314.4 mPa∗s), whilst the T + *Gl*-1 dough had higher viscosity (360.7 mPa∗s). The highest viscosity was found in the T + *Gl*-2 dough (370.7 mPa∗s). Maize doughs containing *Gl* extracts had significantly higher viscosity than the control maize dough (TC). These viscosity values were lower than those reported previously [3], in which ground maguey mushrooms (*P. agaves*) were added to the nixtamalised maize flour. Similarly, Cornejo-Villegas et al. [31] reported that the addition of 2% and 4% of nopal flour increased the viscosity of the maize flour. Interesting relationships were observed amongst moisture content, viscosity and texture in tlayudas. Higher moisture content resulted in greater viscosity and harder texture of the final product. T + *Gl*-1 and T + *Gl*-2 doughs had significantly higher moisture than the TC dough, as well as significantly higher viscosity. It was evident that the T + *Gl*-2 dough had higher viscosity than the T + *Gl*-1 dough. Similarly, T + *Gl*-2 tlayudas showed higher cutting force than T + *Gl*-1 tlayudas, being both higher than TC tlayudas. Relationships between moisture content and viscosity in hybrid maize dough has previously been reported [32], showing that decreasing moisture content reduced intermolecular forces within the dough and increased biopolymer chain mobility, resulting in decreased viscosity. The temperature for dough preparation is a critical factor for differences between moisture and viscosity values [32]; in this study, we used a high temperature of nixtamalisation (87°C) leading to lower moisture and viscosity of doughs, compared with other studies. Future research work can be focused on how the composition of *Gl*-1 or *Gl*-2 extracts affects moisture, viscosity and texture of doughs in the elaboration of tlayudas.

#### 3.2.2. Texture Analysis of Tlayudas

**Figure 2:**
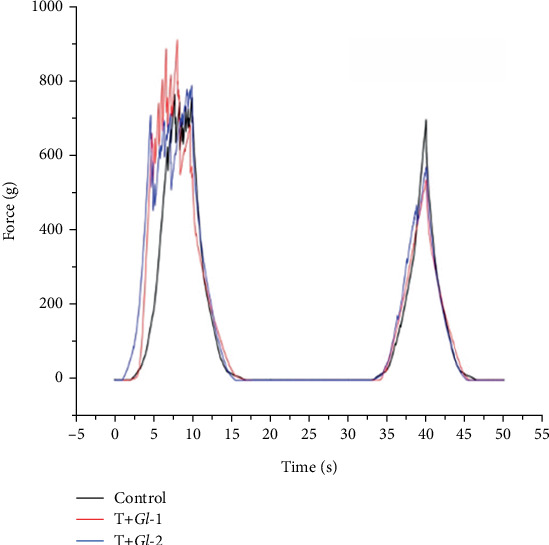
Texture profile analysis (TPA) of tlayudas. TC = tlayudas control. T + *Gl*-1 = tlayudas containing 5 mL of the *Gl*-1 extract (25 mg/mL). T + *Gl*-2 = tlayudas containing 5 mL of the *Gl*-2 extract (25 mg/mL).

The cutting force (i.e. necessary force to perforate tlayudas) was determined in the centre and at the ends of tlayudas from TC, T + *Gl*-1 and T + *Gl*-2 experimental samples (Figure 2). There were significant differences in the cutting force in the centre (g force) between the TC sample (440.50) and the other samples (T + *Gl*-1: 1260.50; T + *Gl*-2: 1369.50), as shown in Table 4. The T + *Gl*-2 sample required a greater cutting force than the T + *Gl*-1 sample. A similar trend was recorded at the ends of tlayudas in the samples studied, TC (976.00), T + *Gl*-1 (1409.50) and T + *Gl*-2 (1450.50), although no significant differences were recorded. García-Rojas et al. [3] also found that tortillas enriched with *P. agaves* required a greater cutting force to be perforated. In terms of TPA, several texture attributes were measured (Table 4). In the fracturability attribute, the significant lowest g force was recorded in the T + *Gl*-1 sample (893.30), compared with the T + *Gl*-2 sample (1038.60). In cohesiveness (Table 4), the T + *Gl*-1 (0.35 g) sample was significantly lower in comparison with TC (0.44 g) and T + *Gl*-2 (0.44 g) samples. In chewiness, there were no significant differences between samples; TC (235.30 g), T + *Gl*-1 (262.09 g) and T + *Gl*-2 (202.75 g) samples also required equivalent force (Table 4).

#### 3.2.3. Colour Determination in Tlayudas

The values of *a*∗ and *b*∗ increased in tlayudas containing *Gl* extracts (Table 4), although no significant differences were recorded. However, the luminosity (*L*∗) decreased significantly in tlayudas containing the *Gl*-1 extract (66.12), compared with the control (TC, 71.98). Similar results were obtained in flatbread made with flour from the edible mushroom *Pleurotus sajor-caju* [33]. Chávez-Santoscoy et al. [4] reported decreased *L*∗, *a*∗ and *b*∗ after adding black bean extract to maize tortillas. There were no significant differences in hue (*h*∗) or chroma (*c*∗) values; tlayudas were yellow hue, close to strict yellow. In Table 4, it can be seen that there were colour differences (Δ*E*∗) between T + *Gl*-1 (8.19 units), T + *Gl*-2 (5.45 units) and the tlayudas control (TC) used as a reference, although no significant differences were found. The colour of products made with nixtamalised maize is derived from physical properties of grains, lime amount used, the thermo-alkaline processing (time and temperature), rest time, nixtamal washing and the final pH [34–36]. Other authors have suggested that, during cooking at high temperatures, the maize dough loses moisture, and there is further gelatinisation of starch, protein denaturing and more intense colours develop due to Maillard reactions [37].

### 3.3. Bromatological Analysis of Tlayudas

Composition analyses of tlayudas are shown in Table 5. Total fat and carbohydrate contents were lower in T + *Gl*-1 and T + *Gl*-2. However, polyunsaturated fat increased 2.3% in T + *Gl*-1 and T + *Gl*-2, compared with TC. This was so for sodium, ash and the moisture content. The calcium content was higher in T + *Gl*-1 (39.6%) and T + *Gl*-2 (29.0%), compared with TC. The highest caloric content was recorded in TC, followed by T + *Gl*-1 and T + *Gl*-2.

Bromatological analysis indicated that tlayudas containing *GI* extracts contain fewer calories, less total fat, less saturated fat and less total carbohydrate compared to control tlayudas. We previously reported a high prevalence of overweight and obesity (82.2%), hypertriglyceridaemia (75.6%) and hypercholesterolaemia (26.7%) in indigenous communities from Oaxaca [38]. As tlayudas are an important staple food widely consumed in these rural regions, we consider that the chemical composition of tlayudas containing *GI* extracts, including important bioactive compounds (glucans and polyphenols), is an important characteristic that may help to mitigate and prevent common public health diseases associated with diet, such as diabetes and cardiovascular pathologies. Tlayudas containing *Gl* extracts showed high calcium content, which is important as the intake of calcium has been reported to be inadequate in the Mexican population, particularly amongst adolescents and adults [39].

### 3.4. Analyses of Bioactive Compounds

#### 3.4.1. Total Polyphenols, Antioxidant Capacity and *β*-Glucans

Tlayudas containing the extracts from *G. lucidum* (T + *Gl*-1: 1027.50 mg GAE/100 g; T + *Gl*-2: 1084.35 mg GAE/100 g) showed higher total polyphenol content in comparison with tlayudas control (TC: 949.48 mg GAE/100 g), although no significant differences were recorded (Table 6). García-Rojas et al. [3] reported that total phenols decreased in maize tortillas enriched with *P. agaves*.

The antioxidant capacity of tlayudas assessed by the ORAC test is shown in Table 6. Antioxidant capacity significantly higher was recorded in tlayudas containing the *Gl*-2 extract (2782.66 TEs/g), in comparison with TC (2387.33 TEs/g) and T + *Gl*-1 (2562.86 TEs/g). A similar trend was previously reported, as the ORAC antioxidant capacity increased from 306.21 to 495.08 *μ*mol TE/g in tortillas enriched with the maguey mushroom [3].

*β*-Glucans increased significantly in tlayudas containing *Gl* extracts (T + *Gl*-1: 0.14%; T + *Gl*-2: 0.24%), in comparison with tlayudas control (TC: 0.12%), as shown in Table 6. A previous report [3] also found that *β*-glucan content increased from 2.17% to 5.80% w/w in tortillas enriched with the maguey mushroom (*P. agaves*).

#### 3.4.2. Analysis of Tlayudas by IR

**Figure 3:**
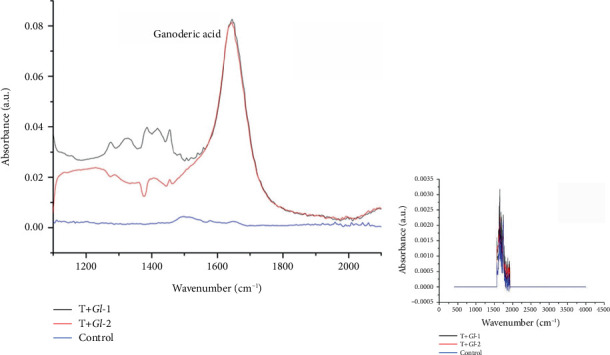
Determination of ganoderic acid A in tlayudas. TC = tlayudas control. T + *Gl*-1 = tlayudas containing 5 mL of the *Gl*-1 extract (25 mg/mL). T + *Gl*-2 = tlayudas containing 5 mL of the *Gl*-2 extract (25 mg/mL). The FTIR spectra for the full range of wavenumber appear on the right as a reference in a smaller image.

The IR spectroscopy results showed the presence of ganoderic acid A in tlayudas containing extracts from *G. lucidum* (T + *Gl*-1; T + *Gl*-2), at the clear strong IR band of 1659 cm^−1^, as reported by Yao et al. [20]. However, the T + *Gl*-1 sample showed a slightly higher absorbance for ganoderic acid A, compared with the T + *Gl*-2 sample (Figure 3). This minor difference may be due to subtle variations in the elaboration process of tlayudas. Although the infrared of the mid-region had a wide range, we show a small region where the ganoderic acid was observed, as there was no other relevant presence in the IR spectrum.

### 3.5. Sensory Evaluation and Acceptability of Tlayudas

In general, we found that tlayudas containing the *Gl*-1 extract (T + *Gl*-1) were better accepted (Table 7), in comparison with tlayudas control (TC) and tlayudas containing the *Gl*-2 extract (T + *Gl*-2). Most affective judges (94%; *n* = 31) liked T + *Gl*-1, so they would be willing to buy it. Similar was the case of tlayudas containing the *Gl*-2 extract (T + *Gl*-2), as 87% of affective judges liked it and would be willing to buy it. The acceptance of tlayudas control (TC) was 84% of affective judges. No significant differences were recorded amongst experimental tlayudas (TC, T + *Gl*-1 and T + *Gl*-2) in the liking level test (9-point hedonic scale), considering all attributes evaluated, excepting colour. Lower scores were recorded in tlayudas control (TC) for appearance (7.16), colour (7.00), smell (6.61), flavour (7.10), texture (7.19) and acceptability (7.13), compared with tlayudas containing *Gl* extracts (Table 7).

### 3.6. Hypolipidaemic Effects of Tlayudas on an In Vivo Model

We studied an in vivo model of hypercholesterolaemia induced by diet administered to C57BL/6 male mice.

#### 3.6.1. Intake and Weight Increase

**Figure 4:**
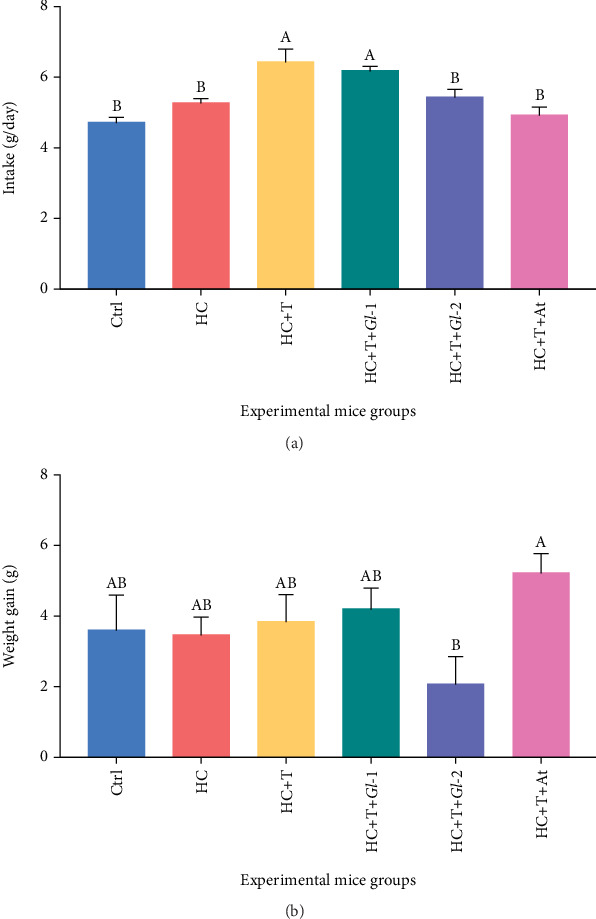
(a) Intake and (b) weight gain from experimental groups studied of C57BL/6 mice administered with a high-cholesterol diet for 45 days. Ctrl: control diet (AIN-93). HC: high-cholesterol diet (0.5% cholesterol). HC + T: high-cholesterol diet (0.5%) + tlayudas control (60%). HC + T + *Gl*-1: high-cholesterol diet (0.5%) + tlayudas containing the *Gl*-1 extract (60%). HC + T + *Gl*-2: high-cholesterol diet (0.5%) + tlayudas containing the *Gl*-2 extract (60%). HC + T + At: high-cholesterol diet (0.5%) + tlayudas control (60%) + atorvastatin drug (0.03 g/100 g). Different letters on columns show statistically significant difference between means (*p* < 0.05; *mean* ± *standard* *error* of the *mean*), according to one-way ANOVA and Tukey's multiple comparisons test.

The average food intake (grammes of diet consumption per day) in the experimental mice groups was as follows: Ctrl (control diet): 4.72, HC (high-cholesterol diet): 5.27, HC + T (high-cholesterol diet + tlayudas control): 6.44, HC + T + *Gl*-1 (high-cholesterol diet + tlayudas containing the *Gl*-1 extract): 6.18, HC + T + *Gl*-2 (high-cholesterol diet + tlayudas containing the *Gl*-2 extract): 5.44 and HC + T + At (high-cholesterol diet + tlayudas control + atorvastatin drug): 4.93. Mice groups showing significantly higher diet consumption, as compared to the other groups (*p* < 0.0001), were HC + T and HC + T + *Gl*-1 (Figure 4). After 45 days, the initial weight (Day 1) of the animals was subtracted from the final weight for obtaining the final weight increase. The results showed that the mice group having the highest significant weight increase was HC + T + At, whereas the lowest significant weight increase was recorded in the HC + T + *Gl*-2 group (*p* = 0.0436). Compared with these groups, the other mice groups did not show significant differences (Figure 4). We observed that tlayudas had good palatability for experimental mice. Meneses et al. [6], using *Gl* extracts directly added to the mice diet (0.5% and 1.0%), did not find significant differences in food intake amongst experimental groups. Regarding weight gain, they also reported the lowest significant value in the mice group consuming the high dose (1.0%) of the *Gl*-2 extract. In this study, mice groups consuming the *Gl*-2 extract showed accordingly the same food intake as Ctrl, HC and HC + T + At, but its weight gain was significantly lower.

#### 3.6.2. Serum Parameters

**Figure 5:**
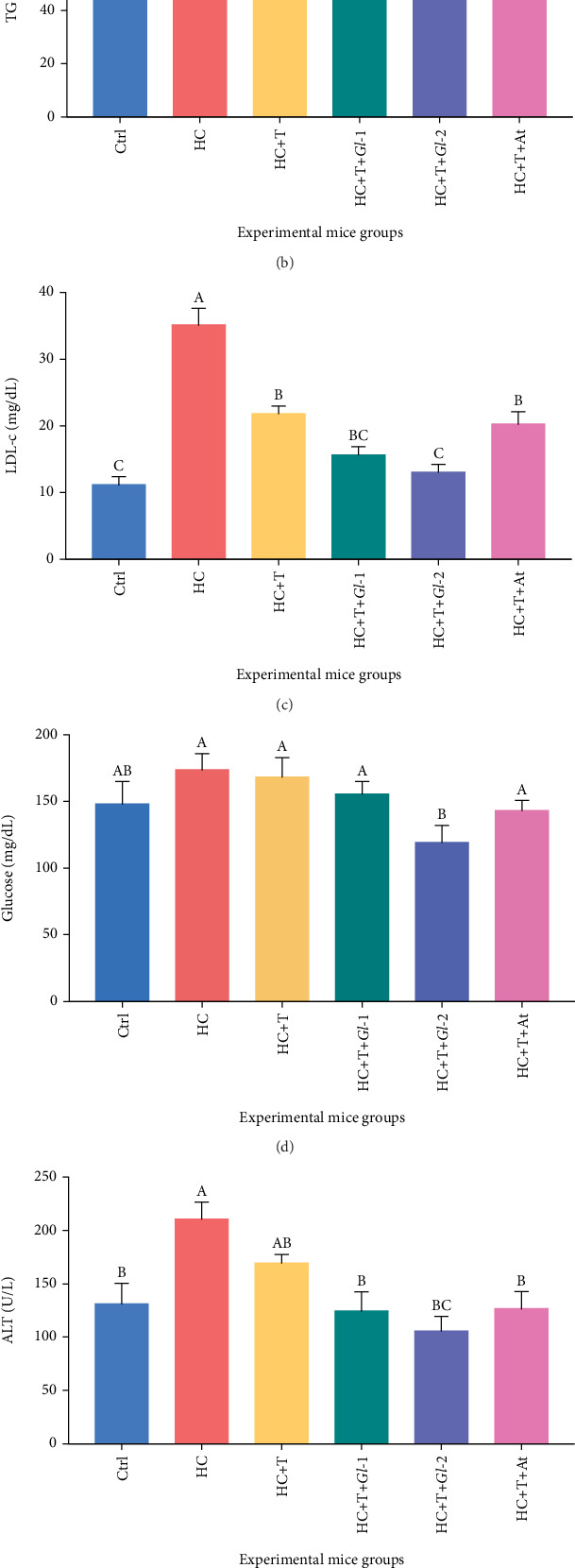
(a) Cholesterol, (b) triglycerides (TG), (c) LDL-c, (d) glucose, (e) ALT and (f) AST parameters in the serum from experimental groups of C57BL/6 mice administered with a high-cholesterol diet for 45 days. Ctrl: control diet (AIN-93). HC: high-cholesterol diet (0.5% cholesterol). HC + T: high-cholesterol diet (0.5%) + tlayudas control (60%). HC + T + *Gl*-1: high-cholesterol diet (0.5%) + tlayudas containing the *Gl*-1 extract (60%). HC + T + *Gl*-2: high-cholesterol diet (0.5%) + tlayudas containing the *Gl*-2 extract (60%). HC + T + At: high-cholesterol diet (0.5%) + tlayudas control (60%) + atorvastatin drug (0.03 g/100 g). Different letters on columns show statistically significant difference between means (*p* < 0.05; *mean* ± *standard* *error* of the *mean*), according to one-way ANOVA and Tukey's multiple comparisons test.

After the consumption of experimental tlayudas for 45 days by C57BL/6 male mice, we observed hypolipidaemic effects of tlayudas containing extracts from *G. lucidum* (T + *Gl*-1 and T + *Gl*-2), as shown in Figure 5. Levels of total cholesterol decreased significantly (*p* = 0.0109) in the HC + T + *Gl*-2 group, in comparison with the HC group; interestingly, no differences with the Ctrl group were recorded. Diet consumption by the HC + T + *Gl*-1 group also showed a tendency to lower cholesterol levels in comparison with the HC group. No significant differences were found between HC + T and HC + T + At mice groups and the HC group. We observed a similar trend in the level of triglycerides; the mice group consuming HC + T + *Gl*-2 showed significantly lower levels (*p* = 0.0453) of triglycerides in comparison with the HC and Ctrl groups. LDL-c levels were significantly lower in experimental groups in comparison with the HC group (*p* < 0.0001). However, the only mice groups that did not have significant differences with the Ctrl group were HC + T + *Gl*-1 and HC + T + *Gl*-2. The level of LDL-c in the HC + T + At group showed no significant differences with the HC + T group, indicating no effect of the atorvastatin drug on this parameter. Glucose levels in the HC + T + *Gl*-2 group were significantly lower (*p* = 0.0306) than those from the HC group. ALT and AST transaminases, indicators of liver dysfunction, were also analysed in mice groups. ALT levels were significantly lower (*p* = 0.0001) in HC + T + *Gl*-1, HC + T + *Gl*-2 and HC + T + At groups in comparison with the HC group, but similar to those from the Ctrl group. AST levels in the HC + T + *Gl*-1 and HC + T + *Gl*-2 groups were significantly lower (*p* = 0.0119) in comparison with the HC group, but equivalent to the Ctrl group.

The results showed the cholesterol-lowering properties of *Gl* extracts integrated into new functional food products, such as tlayudas (T + *Gl*-1 and T + *Gl*-2), administered to an in vivo model of hypercholesterolaemia. The heat treatment (*ca*. 400°C for 6.40 min) of the tlayudas cooking process did not affect the hypocholesterolaemic properties of *G. lucidum* extracts [6–8, 16]. Triglycerides and LDL-c also decreased after the consumption of extracts from *G. lucidum*. The mechanism of action of these hypolipidaemic effects is linked to gene expression associated with cholesterol metabolism, decreased lipogenesis (Srebp1c, Acaca and Fasn genes) and reverse cholesterol transport (abcg5 and abcg8 genes), involving the inhibition of the rate-limiting enzyme for cholesterol biosynthesis (Hmgcr, 3-hydroxy-3-methylglutaryl-CoA reductase) and important prebiotic effects on the gut microbiota [6–8, 16]. The hypolipidaemic properties of *G. lucidum* are based on the synergistic action of its bioactive compounds, including *α*-glucans, *β*-glucans, triterpenes and polyphenols. Glucans increase viscosity in the intestine, leading to decreased lipid absorption, and exert prebiotic effects, increasing bacteria, such as *Lactobacillus*, that are inversely related to plasma lipid levels [6, 7]. Triterpenoids from *G. lucidum* showed inhibitory capacity on the Hmgcr rate-limiting enzyme [40]. Alcohol consumption has previously been linked to increased ALT and AST transaminases, which are indicators of liver damage including the development of fatty liver [41]. In the present study, we found that experimental mice groups consuming tlayudas containing hydroalcoholic *Gl* extracts had similar ALT and AST values, which were not significantly different from the Ctrl group but were significantly lower than those from the mice group consuming a high-cholesterol diet. Moreover, we previously showed that *Gl* extracts used in this study are not toxic through an oral toxicity test of repeated doses even at extreme high concentrations of 5000 mg/kg weight [16]. It is also important to mention that the added hydroalcoholic solvent is evaporated and eliminated during the traditional cooking process of tlayudas (400°C). Low concentrations of hydroalcoholic *Gl* extracts contained in tlayudas studied do not cause adverse effects in the liver associated with transaminase levels.

## 4. Conclusion

This study revealed that standardised hydroalcoholic extracts of a Mexican genotype of the medicinal mushroom *G. lucidum* can be added to tlayudas, affecting positively their physical properties. The new functional food products, tlayudas containing extracts from *G. lucidum* (either *Gl*-1 or *Gl*-2), are more resistant and have better sensory attributes. Functional properties also improved significantly, increasing antioxidant activity, the content of *β*-glucans and ganoderic acid A (an important triterpenoid from *G. lucidum*), and several nutritional components. The heat treatment (*ca*. 400°C for 6.40 min) of the tlayudas cooking process did not affect *G. lucidum* compounds, remaining stable and bioactive in the food matrix. The content of *β*-glucans increased up to 100% (T + *Gl*-2) in tlayudas containing *Gl* extracts, the ORAC antioxidant capacity up to 16.5% (T + *Gl*-2) and the total polyphenols up to 14.2% (T + *Gl*-2). The consumption of tlayudas containing extracts of *G. lucidum* (T + *Gl*-1 or T + *Gl*-2) decreased serum levels of cholesterol, triglycerides and LDL-c in C57BL/6 mice groups, as well as levels of glucose and transaminases. Tlayudas containing the *Gl*-2 extract (T + *Gl*-2) decreased serum lipids and glucose levels further than the atorvastatin drug. These new functional food products of tlayudas (T + *Gl*-1 and T + *Gl*-2) with hypolipidaemic properties, derived from a highly consumed and low-cost traditional Mexican food, could be used to promote healthier diets for preventing cardiovascular and chronic degenerative diseases (dyslipidaemias, atherosclerosis, diabetes, cancer and metabolic syndrome, amongst others) in target populations. However, further studies are needed on gene expression analysis in mice tissues in order to understand the mechanisms of action of bioactive compounds at the molecular level. Interesting research work can also be developed about the positive prebiotic effects of tlayudas plus *Gl* extracts on the gut microbiota using in vivo models or clinical trials.

## Figures and Tables

**Table 1:** Composition of six diets consumed by the experimental groups of C57BL/6 mice studied.

**Ingredients (g/kg)**	**Ctrl**	**HC**	**HC + T**	**HC + T + *Gl*-1**	**HC + T + *Gl*-2**	**CH + T + At**
l-Cystine	3.0	3.0	1.2	1.2	1.2	1.2
Choline	2.5	2.5	1	1	1	1
Vitamin mix	10.0	10.0	4	4	4	4
Cellulose	50.0	50.0	21.98	21.98	21.98	21.98
Mineral mix	35.0	35.0	14	14	14	14
Soybean oil	70.0	70.0	28	28	28	28
Cornstarch	397.5	397.5	159	159	159	159
Dextrinised cornstarch	132.0	132.0	52.8	52.8	52.8	52.8
Sucrose	100.0	100.0	40	40	40	40
Casein	200.0	200.0	80	80	80	80
Cholesterol	—	5.0	5.0	5.0	5.0	5.0
Atorvastatin	—	—	—	—	—	0.3
Tlayudas control	—	—	600	—	—	600
Tlayudas *Gl*-1			—	600	—	—
Tlayudas *Gl*-2	—	—	—	—	600	—

*Note:* Differences in the amount of ingredients of the diet from the experimental HC group in comparison with the other experimental treated groups are due to adjustments made by the integration of 60% of tlayudas, a normal proportion traditionally consumed by rural communities.

Abbreviations: Ctrl, control diet (AIN-93); HC, high-cholesterol diet (0.5% cholesterol); HC + T, high-cholesterol diet (0.5%) + tlayudas control (60%); HC + T + At, high-cholesterol diet (0.5%) + tlayudas control (60%) + atorvastatin drug (0.03 g/100 g); HC + T + *Gl*-1, high-cholesterol diet (0.5%) + tlayudas containing the *Gl*-1 extract (60%); HC + T + *Gl*-2, high-cholesterol diet (0.5%) + tlayudas containing the *Gl*-2 extract (60%).

**Table 2:** Physical properties of the local organic maize grain from Matatlán, Oaxaca, Mexico.

**FI (%)**	**HGW (g)**	**MG (%)**	**MN (%)**	**Colour**		
				**L**∗	**h**∗	**c**∗
38.0 ± 3.21	46.3 ± 1.26	11.9 ± 0.34	43.9 ± 1.16	68.6 ± 2.2	81.1 ± 1.1	30.0 ± 1.5

*Note:* Average from five replicates.

Abbreviations: *c*∗, chroma; FI, flotation index; *h*∗, hue; HGW, hundred grain weight; *L*∗, luminosity; MG, moisture of grain on a wet basis; MN, moisture of nixtamal on a dry basis.

**Table 3:** Physical properties of the nixtamalised maize doughs prepared for the elaboration of tlayudas studied.

**Sample**	**MD (%)**	**VD (mPa∗s)**
TC	58.2 ± 0.8^*b*^	314.4 ± 1.2^*c*^
T + *Gl-*1	58.7 ± 0.07^*a*^	360.7 ± 0.8^*b*^
T + *Gl-*2	58.7 ± 0.10^*a*^	370.7 ± 0.4^*a*^

*Note:* Average from five replicates. Means in a column showing differing letters indicate statistically significant difference, *p* < 0.05. Values correspond to one-way ANOVA from the Tukey's multiple comparisons test.

Abbreviations: MD, moisture in doughs; TC, tlayudas control; T + *Gl*-1, tlayudas containing 5 mL of the *Gl*-1 extract (25 mg/mL); T + *Gl*-2, tlayudas containing 5 mL of the *Gl*-2 extract (25 mg/mL); VD, viscosity in doughs.

**Table 4:** Physical properties of tlayudas studied.

**Variables**		**Tlayudas samples**		
		**TC**	**T + *Gl*-1**	**T + *Gl*-2**
Cutting force	Centre (g force)	440.50 ± 183.14^*b*^	1260.50 ± 149.20^*a*^	1369.50 ± 120.92^*a*^
	Ends (g force)	976.00 ± 107.48^*a*^	1409.50 ± 340.12^*a*^	1450.50 ± 188.80^*a*^

TPA	Fracturability (g force)	970.59 ± 21.54^*ab*^	893.30 ± 14.76^*b*^	1038.60 ± 48.82^*a*^
	Cohesiveness (g)	0.44 ± 0.01^*a*^	0.35 ± 0.01^*b*^	0.44 ± 0.03^*a*^
	Chewiness (g)	235.30 ± 3.09^*a*^	262.09 ± 9.25^*a*^	202.75 ± 29.25^*a*^

Colour	*L*∗	71.98 ± 1.85^*a*^	66.12 ± 0.29^*b*^	67.81 ± 0.14^*ab*^
	*a*∗	1.78 ± 0.09^*a*^	3.72 ± 0.19^*a*^	2.41 ± 0.08^*a*^
	*b*∗	20.60 ± 0.14^*a*^	25.78 ± 0.19^*a*^	23.32 ± 0.21^*a*^
	*h*∗	85.17 ± 2.06^*a*^	82.29 ± 5.17^*a*^	84.08 ± 1.05^*a*^
	*c*∗	20.68 ± 1.91^*a*^	26.12 ± 4.09^*a*^	23.45 ± 0.51^*a*^
	Δ*E*∗	-∗	8.19 ± 3.68^*a*^	5.45 ± 2.21^*a*^

*Note:* Average from five replicates. Different letters in rows show statistically significant difference between means (*p* < 0.05), according to one-way ANOVA and Tukey's multiple comparisons test. ∗ = Parameter used as control.

Abbreviations: *c*∗, chroma; *h*∗, hue; *L*∗, luminosity; TC, tlayudas control; T + *Gl*-1, tlayudas containing 5 mL of the *Gl*-1 extract (25 mg/mL); T + *Gl*-2, tlayudas containing 5 mL of the *Gl*-2 extract (25 mg/mL); TPA, texture profile analysis.

**Table 5:** Bromatological analysis of tlayudas studied.

**Analysis (100 g final product)**	**TC**	**T + *Gl*-1**	**T + *Gl*-2**
Calories (kcal)	378	377	371
Calories from fat	49	48	43
Total fat (g)	5.40	5.38	4.81
Saturated fat (%)	18	17	17
Monounsaturated fat (%)	39	39	39
Polyunsaturated fat (%)	43	44	44
Trans fat (%)	0	0	0
Cholesterol (mg)	0	0	0
Moisture (g)	6.40	7.21	7.95
Ash (g)	1.47	1.59	1.56
Sodium (mg)	9.94	12.8	13.0
Total carbohydrate (g)	77.83	77.13	77.07
Dietary fibre (g)	8.8	7.3	7.7
Total sugars (g)	0.6	0.6	0.6
Added sugars (g)	0.6	0.6	0.6
Protein (g)	8.90	8.69	8.60
Calcium (mg)	179	250	231
Iron (mg)	3.33	3.29	3.02
Potassium (mg)	277	278	273
Vitamin D (*μ*g)	0	0	0

Abbreviations: TC, tlayudas control; T + *Gl*-1, tlayudas containing 5 mL of the *Gl*-1 extract (25 mg/mL); T + *Gl*-2, tlayudas containing 5 mL of the *Gl*-2 extract (25 mg/mL).

**Table 6:** The content of bioactive compounds in tlayudas studied.

**Sample**	**Total polyphenols (mg GAE/100 g of tlayudas)**	**ORAC (trolox equivalents/g of tlayudas)**	***β*-glucans (% w/w)**
TC	949.48 ± 37.12^*a*^	2387.33 ± 9.23^*c*^	0.12 ± 0.01^*c*^
T + *Gl-*1	1027.50 ± 70.51^*a*^	2562.86 ± 48.60^*b*^	0.14 ± 0.01^*b*^
T + *Gl-*2	1084.35 ± 61.33^*a*^	2782.66 ± 31.23^*a*^	0.24 ± 0.005^*a*^

*Note:* Average of five replicates. Different letters in columns show statistically significant difference between means (*p* < 0.05), according to one-way ANOVA and Tukey's multiple comparisons test.

Abbreviations: TC, tlayudas control; T + *Gl*-1, tlayudas containing 5 mL of the *Gl*-1 extract (25 mg/mL); T + *Gl*-2, tlayudas containing 5 mL of the *Gl*-2 extract (25 mg/mL).

**Table 7:** Average data of sensory attributes from tlayudas containing standardised extracts from *Ganoderma lucidum*.

**Sample**	**Attributes**					
	**Appearance**	**Colour**	**Smell**	**Flavour**	**Texture**	**Acceptability**
TC	7.16 ± 1.29^*a*^	7.00 ± 1.51^*b*^	6.61 ± 1.26^*a*^	7.10 ± 1.22^*a*^	7.19 ± 1.30^*a*^	7.13 ± 1.36^*a*^
T + *Gl*-1	7.71 ± 0.94^*a*^	7.77 ± 0.88^*a*^	7.26 ± 1.32^*a*^	7.58 ± 1.29^*a*^	7.87 ± 1.12^*a*^	7.77 ± 0.92^*a*^
T + *Gl*-2	7.39 ± 1.36^*a*^	7.39 ± 1.20^*ab*^	7.26 ± 1.34^*a*^	7.42 ± 1.39^*a*^	7.23 ± 1.28^*a*^	7.35 ± 1.28^*a*^

*Note:* Affective judges: 31. Means in a column showing differing letters indicate statistically significant difference, *p* < 0.05. Values correspond to one-way ANOVA from Tukey's multiple comparisons test.

Abbreviations: TC, tlayudas control; T + *Gl*-1, tlayudas containing 5 mL of the *Gl*-1 extract (25 mg/mL); T + *Gl*-2, tlayudas containing 5 mL of the *Gl*-2 extract (25 mg/mL).

## Data Availability

All relevant data are within the manuscript and its supporting information file.

## Nomenclature

ALT: alanine transaminase
AST: aspartate aminotransferase
a.u.: arbitrary units
FI: flotation index
*Gl*: *Ganoderma lucidum*
*Gl*-1: extracts from *Gl* basidiocarps cultivated on the control substrate
*Gl*-2: extracts from *Gl* basidiocarps cultivated on substrate treated with acetylsalicylic acid (ASA, 10 mM)
HGW: hundred grain weight
IR: infrared spectroscopy
MD: moisture in doughs
MG: moisture of grain
MN: moisture of nixtamal
LDL-c: low-density lipoprotein cholesterol
ORAC: antioxidant capacity by oxygen radical absorbance capacity
TC: *tlayudas* *control* = 100 *g* of *control* *traditional* *maize* *dough* + 5 *mL* of *water*
TPA: texture profile analysis
T + *Gl*-1: *tlayudas* *Gl* − 1 = 100 *g* of *traditional* *maize* *dough* + 5 *mL* of the *Gl* − 1 *extract* (25 *mg*/*mL*)
T + *Gl*-2: *tlayudas* *Gl* − 2 = 100 *g* of *traditional* *maize* *dough* + 5 *mL* of the *Gl* − 2 *extract* (25 *mg*/*mL*)
VD: viscosity in doughs

## Supporting Information

Supporting Information Additional supporting information can be found online in the Supporting Information section. Table S1: Chemical composition and analysis of standardised extracts from mature basidiomata of Mexican *Ganoderma lucidum*, cultivated on *Quercus* sawdust (*Gl*-1) and *Quercus* sawdust plus acetylsalicylic acid (ASA 10 mM; *Gl*-2) [6, 7, 16].
