# Supplementary material for: Characterisation and Hypolipidaemic Effects of Tlayudas, Widely Consumed Tortillas, Containing Ganoderma lucidum Extracts on an In Vivo Model of Hypercholesterolaemia
Source: Int J Food Sci. 2025 Jul 29;2025:8096060. doi: 10.1155/ijfo/8096060 (PMC12324917; doi:10.1155/ijfo/8096060)
Supplement: Supporting Information — Additional supporting information can be found online in the Supporting Information section. Table S1: Chemical composition and analysis of standardised extracts from mature basidiomata of Mexican Ganoderma lucidum, cultivated on Quercus sawdust (Gl-1) and Quercus sawdust plus acetylsalicylic acid (ASA 10 mM; Gl-2) [6, 7, 16]. [file 8096060.f1.DOCX]

**Supplementary Table 1**: Chemical composition and analysis of standardized extracts from mature basidiomata of Mexican *Ganoderma lucidum*, cultivated on *Quercus* sawdust (*Gl*-1) and *Quercus* sawdust plus acetylsalicylic acid (ASA 10 mM; *Gl*-2) [6,16].

| **Analysis** | ***Gl*-1** | ***Gl*-2** | **Unit** | **Method** | **Technique** |
| --- | --- | --- | --- | --- | --- |
| Total protein | 0.365 | 0.315 | % | AOAC. 1995. No. 991.20 | Kjeldahl method |
| Carbohydrates by difference^a^ | 0.58 | 0.58 | % | Merrill & Watt. 1973 | - |
| Glucose | 0.1 | 0.1 | % | AOAC. 1995.  No. 996.04 | Ion chromatography |
| Total sugars | 0.1 | 0.1 | % | AOAC. 1995.  No. 996.04 | Ion chromatography |
| Calories^a^ | 4 | 4 | /100 g | Methods of Analysis for Nutritional Labelling, AOAC International. 1993 | - |
| Total dietary fiber | 0.15 | 0.10 | % | AOAC. 2003.  No. 985.29 | Gravimetric analysis |
| Total glucans | 15.96 | 17.01 | % w/w | AOAC. No. 995.16 | Colorimetric method |
| α-glucans | 14.19 | 15.14 | % w/w | AOAC. No. 995.16 | Colorimetric method |
| β-glucans | 1.77 | 1.87 | % w/w | AOAC. No. 995.16 | Colorimetric method |
| Total polyphenols | 2.185 | 1.858 | mg GAE/g | Folin Ciocalteau assay | Colorimetric method |
| Reactive species | 350,811.50 | 376,117.06 | μmol of Trolox Equivalent/g | Oxygen radical absorbance capacity (ORAC) assay | Fluorescence assay |
| Fat | 0.01 | 0.01 | % | AOAC. 2000. 989.05 | Gravimetric analysis |
| Ash | 0.10 | 0.10 | % | AOAC. 2000.  No. 942.05 | Gravimetric analysis |
| Vitamin B1 (thiamine) | 0.03 | 0.025 | mg/100 g | R-Biopharm. 2011. VitaFast Vitamin B1 | Microbiological assay |
| Vitamin B2 (riboflavin) | 0.04 | 0.035 | mg/100 g | R-Biopharm. 2011. VitaFast Vitamin B2 | Microbiological assay |
| Vitamin B3 (niacin) | 0.47 | 0.42 | mg/100 g | R-Biopharm. 2011. VitaFast Vitamin B3 | Microbiological assay |
| Vitamin B6 (pyridoxin) | 0.01 | 0.01 | mg/100 g | R-Biopharm. 2011. VitaFast Vitamin B6 | Microbiological assay |
| Vitamin B12 (cyanocobalamin) | 0.05 | 0.045 | µg/100 g | R-Biopharm. 2011. VitaFast Vitamin B12 | Microbiological assay |
| Vitamin D | <2.0 | <2.0 | IU/100 g | AOAC. 2009.  No. 982.29 | HPLC |
| Calcium | 1.15 | 1.10 | mg/100 g | AOAC. 2005.  No. 974.14 | Atomic absorption spectrometry |
| Copper | 120 | 115 | Ppb | Method CFAN/ORS/DBC/CHCB. FDA 2011 | ICP-MS |
| Iron | 200 | 335 | Ppb | Method CFAN/ORS/DBC/CHCB. FDA 2011 | ICP-MS |
| Magnesium | 2.35 | 2.25 | mg/100 g | AOAC. 2005.  No. 974.14 | Atomic absorption spectrometry |
| Manganese | 120 | 135 | Ppb | Method CFAN/ORS/DBC/CHCB. FDA 2011 | ICP-MS |
| Phosphorus | 10.5 | 12.5 | mg/100 g | AOAC. 2007.  No. 970.39 | Spectrophotometric method |
| Potassium | 36.5 | 32.5 | mg/100 g | AOAC. 2005.  No. 974.14 | Atomic absorption spectrometry |
| Selenium | <100 | <100 | Ppb | Method CFAN/ORS/DBC/CHCB. FDA 2011 | ICP-MS |
| Sodium | 1.9 | 1.85 | mg/100 g | AOAC. 2005.  No. 974.14 | Atomic absorption spectrometry |
| Zinc | 450 | 405 | Ppb | Method CFAN/ORS/DBC/CHCB. FDA 2011 | ICP-MS |
| Organic acid profile: |  |  |  |  |  |
| Acetic acid | <0.001 | <0.001 | % | AOAC. 2000.  No. 986.13 | HPLC |
| Butyric acid | <0.001 | <0.001 | % | AOAC. 2000.  No. 986.13 | HPLC |
| Citric acid | 0.0065 | 0.0060 | % | AOAC. 2000.  No. 986.13 | HPLC |
| Fumaric acid | 0.002 | 0.002 | % | AOAC. 2000.  No. 986.13 | HPLC |
| Gluconic acid | <0.001 | <0.001 | % | AOAC. 2000.  No. 986.13 | HPLC |
| Lactic acid | <0.001 | <0.001 | % | AOAC. 2000.  No. 986.13 | HPLC |
| Malic acid | 0.0175 | 0.0150 | % | AOAC. 2000.  No. 986.13 | HPLC |
| Oxalic acid | 0.003 | 0.003 | % | AOAC. 2000.  No. 986.13 | HPLC |
| Propionic acid | <0.001 | <0.001 | % | AOAC. 2000.  No. 986.13 | HPLC |
| Quinic acid | <0.001 | <0.001 | % | AOAC. 2000.  No. 986.13 | HPLC |
| Succinic acid | <0.001 | <0.001 | % | AOAC. 2000.  No. 986.13 | HPLC |
| Tartaric acid | <0.001 | <0.001 | % | AOAC. 2000.  No. 986.13 | HPLC |
|  |  |  |  |  |  |
| Crude fiber | 0 | 0 | % | AOAC. 1990.  No. 962.09 | Gravimetric analysis |
| Fructose | 0 | 0 | % | AOAC. 1995.  No. 996.04 | Ion chromatography |
| Lactose | 0 | 0 | % | AOAC. 1995.  No. 996.04 | Ion chromatography |
| Maltose | 0 | 0 | % | AOAC. 1995.  No. 996.04 | Ion chromatography |
| Sucrose | 0 | 0 | % | AOAC. 1995.  No. 996.04 | Ion chromatography |
| Vitamin A | 0 | 0 | IU/100 g | AOAC. 2001.  No. 292.13 | HPLC |
| Vitamin B9  (folic acid) | 0 | 0 | µg/100 g | R-Biopharm. 2011. VitaFast Folic Acid | Microbiological assay |
| Vitamin C | 0 | 0 | mg/100 g | Brause *et al*. 2003 | HPLC |
| Vitamin E | 0 | 0 | mg/100 g | AOAC. 2000.No. 992.03 | HPLC |
| Retinol | 0 | 0 | IU/100 g | AOAC. 2002.  No. 938.04/2001.13 | HPLC |
| Total omega-3 | n/a | n/a | mg/100 g sample | AOCS. 2002.  No. Ce 1f-96 | Ion chromatography |
| Total monounsaturated fat | n/a | n/a | g/100 g sample | AOCS. 2002.  No. Ce 1f-96 | Ion chromatography |
| Total polyunsaturated fat | n/a | n/a | g/100 g sample | AOCS. 2002.  No. Ce 1f-96 | Ion chromatography |
| Total saturated fat | n/a | n/a | g/100 g sample | AOCS. 2002.  No. Ce 1f-96 | Ion chromatography |
| Total trans fat | n/a | n/a | g/100 g sample | AOCS. 2002.  No. Ce 1f-96 | Ion chromatography |
| Alpha linolenic (ALA) | n/a | n/a | mg/100 g sample | AOCS. 2002.  No. Ce 1f-96 | Ion chromatography |
| Docosahexaenoic (DHA) | n/a | n/a | mg/100 g sample | AOCS. 2002.  No. Ce 1f-96 | Ion chromatography |
| Docosapentaenoic | n/a | n/a | mg/100 g sample | AOCS. 2002.  No. Ce 1f-96 | Ion chromatography |
| Docosatrienoic | n/a | n/a | mg/100 g sample | AOCS. 2002.  No. Ce 1f-96 | Ion chromatography |
| Eicosapentaenoic (w-3) (EPA) | n/a | n/a | mg/100 g sample | AOCS. 2002.  No. Ce 1f-96 | Ion chromatography |
| Eicosatrienoic (11,14,17) | n/a | n/a | mg/100 g sample | AOCS. 2002.  No. Ce 1f-96 | Ion chromatography |
|  |  |  |  |  |  |
| AOAC= Official methods of analysis of the Association of Official Analytical Chemists, U.S.A. | | | | | |
| AOCS= Official methods of analysis of the American Oil Chemists’ Society, U.S.A.  HPLC= High-performance liquid chromatography.  ICP-MS= Inductively coupled plasma-mass spectrometry. | | | | | |
| ^a^ Calculated data based on specific standards.  n/a= Not available. | | | | | |
|  | | | | | |
